# Obesity as a risk factor for multiple myeloma: insight on the role of adipokines

**DOI:** 10.3389/pore.2023.1611338

**Published:** 2023-08-10

**Authors:** Wenting Tie, Tao Ma, Zhigang Yi, Jia Liu, Yanhong Li, Jun Bai, Lijuan Li, Liansheng Zhang

**Affiliations:** ^1^ Department of Hematology, Lanzhou University Second Hospital, Lanzhou, China; ^2^ Department of Endocrinology, Lanzhou University Second Hospital, Lanzhou, China; ^3^ Department of Hematology, The Affiliated Hospital of Southwest Medical University, Luzhou, China

**Keywords:** multiple myeloma, adipokine, obesity, adiponectin, leptin

## Abstract

Multiple myeloma (MM) is a hematologic disorder characterized by the accumulation of malignant plasma cells in the bone marrow. Genetic and environmental factors are contributed to the etiology of MM. Notably, studies have shown that obesity increases the risk of MM and worsens outcomes for MM patients. Adipokines play an important role in mediating the close association between MM and metabolic derangements. In this review, we summarize the epidemiologic studies to show that the risk of MM is increased in obese. Accumulating clinical evidence suggests that adipokines could display a correlation with MM. *In vitro* and *in vivo* studies have shown that adipokines are linked to MM, including roles in the biological behavior of MM cells, cancer-associated bone loss, the progression of MM, and drug resistance. Current and potential therapeutic strategies targeted to adipokines are discussed, proposing that adipokines can guide early patient diagnosis and treatment.

## Introduction

Multiple myeloma (MM) is a blood cancer of abnormal clonal plasma cells in the bone marrow. MM accounts for 1% of neoplastic diseases and is the second most common hematological malignancy that commonly affects older adults (median age at diagnosis is 69 years) [1, 2]. Patients often suffer from anemia, kidney injury, bone destruction, and hypercalcemia. MM develops from the premalignant state monoclonal gammopathy of undetermined significance (MGUS) and smoldering multiple myeloma (SMM). To diagnose MM, clinical symptoms are used, and the monoclonal proteins in blood and urine need to be detected. Clinical symptoms are used to diagnose MM, and the monoclonal proteins in blood and urine must be detected. Autologous stem-cell transplantation (ASCT), proteasome inhibitors, immunomodulatory drugs, and monoclonal antibodies have improved outcomes with MM. Although therapeutic advances made in the past few years have led to improved outcomes and longer survival, MM remains incurable. Therefore, it is of great significance to study the etiology and pathogenesis of myeloma to find new therapeutic targets and improve the survival rate of patients.

Although little is known about the etiology of MM, genetic, antigenic stimulation, and environmental factors are believed to contribute to MM onset and progression [3]. Some risk factors are associated with MM, such as obesity, male sex, age, chronic inflammation, and dioxin exposure [2]. Accumulating evidence suggests that obesity plays a critical role in the risk of developing MM, and the increased body mass index (BMI) has been linked to the progression and higher mortality [4–6]. The bone marrow microenvironment plays a vital role in the proliferation, survival, progression, and drug resistance of MM cells [2]. The bone marrow microenvironment is composed of cellular and non-cellular compartments. The cellular compartment comprises MM cells, lymphocytes, natural killer cells, macrophages, monocytes, dendritic cells, osteoclasts, osteoblasts, and adipocytes. And the non-cellular compartment is made up of extracellular matrix proteins, adhesion molecules, cytokines, and growth factors. Bone marrow adipocytes are the main component of the bone marrow microenvironment [7]. Bone marrow adipocytes in close contact with bone cells, hematopoietic cells, and immune cells are considered within bone metabolism, hematopoiesis, cancer, and systemic energy metabolism. Adipocytes found in bone marrow have important connections with bone cells, hematopoietic cells, and immune cells. These connections play significant roles in bone metabolism, hematopoiesis, cancer, and systemic energy metabolism. Using the C57BL/KaLwRij murine model of myeloma, mice were inoculated with 5TGM1-GFP cells or PBS control by intravenous injection. Bone marrow adipocytes were found to be increased in early-stage myeloma [8]. MM cell lines (RPMI-8226 and NCI-H929) were cocultured with adipocytes and showed greater capabilities of proliferation and adhesion [9]. These findings indicate that bone marrow adipocytes promote the proliferation of MM cells.

Obesity is closely related to the onset of many types of malignancies [10]. The mechanisms linking obesity to cancer involve insulin resistance, abnormalities of the IGF-1 axis, inappropriate sex hormone secretion, inflammation and oxidative stress, adipokines action, microenvironment perturbations, and the altered intestinal microbiome [11]. In obesity, adipose tissue hypoxia triggers alternations of adipokines levels altering adipokines levels, may be associated with the progression of various cancers [12–14]. This review critically summarizes the rapidly expanding field of bone marrow adipocytes as an endocrine organ and how they communicate with MM cells through the secretion of adipokines. First, we will briefly address the changes in adipokines discuss the changes in adipokines of MM. Second, we will present the biological effect of adipokines. Finally, we will elaborate on the adipokines associated with MM progression as growth, proliferation, and drug resistance, including findings from *in vitro*, *in vivo*, and human studies, and therapeutic strategy target to adipokines.

### Adipokines in MM: friend or foe?

**FIGURE 1:**
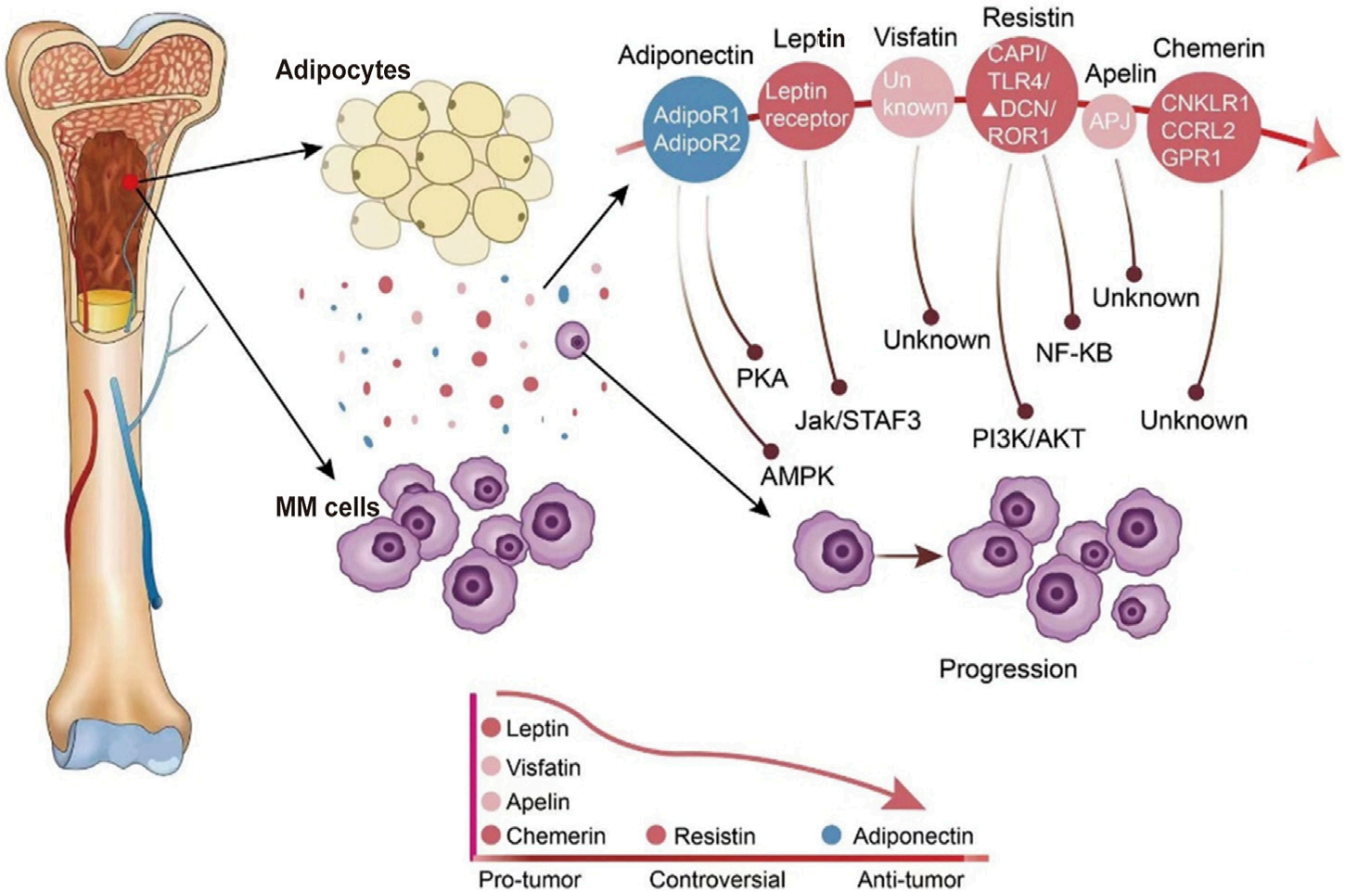
The expression of adipokines in the bone marrow environment and adipokines modify the behavior of MM cells. Adipocytes in the bone marrow secrete a variety of different adipokines. The expression of adipokines in the bone marrow microenvironment of MM patients is indicated by different colors, with blue indicating reduced secretion and red indicating increased secretion.

Adipose tissue was initially thought of as a fat depot but is now widely recognized as an endocrine organ that secretes numerous peptide factors called adipokines. They play an essential role in metabolic diseases and neoplasms in paracrine and endocrine. They can regulate glucose and lipid metabolism, inflammation, and immune response [15]. After the first adipokine leptin was discovered, over 600 adipokines have been discovered and studied [16]. Some adipokines stimulate cancer progression through oncogenic signaling or indirect mechanisms, such as angiogenesis and immunomodulation [17], while others with oncogenic effects have their expression suppressed in malignancies. Therefore, alterations in adipokines may affect the processes and the immune responses of cancers. The role of adipokines in MM is comparatively less known. A selected group of adipokines with demonstrated roles in MM are reviewed below (Figure 1).

#### Adiponectin

Adiponectin is a 28 KDa protein secreted by adipose tissue, which is one of the most widely studied adipokines [18]. Besides the adipose tissue, adiponectin is also produced to some extent by bone marrow [19]. Adiponectin has two major receptors, AdipoR1 and AdipoR2. A third receptor had been isolated, identical to a unique cadherin molecule [20, 21]. Adiponectin binds to receptors to participate in insulin-sensitizing, lipid metabolism, energy regulation, inflammation, and cancer development [22]. Circulating levels of adiponectin show an inverse correlation with multiple diseases, such as obesity, type 2 diabetes, atherosclerosis, hypertension, dyslipidemia, and nonalcoholic fatty liver disease [23–27]. It also negatively correlates with several cancers, one of which is MM [28, 29]. Thus adiponectin has generally been considered a beneficial adipokine.

In a pooled investigation of 624 MM patients and 1,246 controls from seven cohorts, Hofmann et al. revealed that serum adiponectin levels were lower among the patients than controls (medians of 11.5 and 12.8 μg/mL, respectively; *p* = 0.001). Furthermore, they stratified the samples according to BMI and observed an inverse association between adiponectin levels and MM risk among overweight (OR = 0.41, CI = 0.26–0.65, *p* < 0.001) or obese subjects (OR = 0.41, CI = 0.17–0.98, *p* ≤ 0.039) [30]. Hofmann et al. investigated the levels of adiponectin in serum samples from 213 patients (84 with MGUS, 104 with SMM, and 25 with MM). They found total adiponectin levels were 16% lower among SMM patients (CI = −31% to 2%) and 20% lower among MM patients (CI = −40% to 7%) compared to patients with MGUS [29]. Furthermore, another study showed decreased levels of high molecular weight adiponectin in MGUS patients who progressed to MM (4.5 ± 0.5 μg/mL) compared to MGUS who did not progress to MM (6.4 ± 1.6 μg/mL). The studies used gene array analysis of bone marrow between KaLwRij mice that permit murine 5T myeloma cells compared to nonpermissive mice. Adiponectin was decreased in the bone marrow of KaLwRij mice [31]. These studies suggested low adiponectin levels may associate with MM, particularly among overweight and obese individuals. And adiponectin may play a protective role in the development of MGUS to SMM/MM. This suggests that adiponectin may have a protective effect in the development of MGUS to SMM/MM. Lower blood levels of adiponectin in MM patients before the start of any treatment suggest that adiponectin represents a potential biomarker at the onset of disease to predict progression.

The mechanisms behind the association between adiponectin and MM are still unclear. Some studies provide insights into the mechanisms of adiponectin in MM. Adiponectin induces the apoptosis and cell cycle arrest of MM cells via activation of protein kinase A (PKA) and increased AMP-activated protein kinase (AMPK) activation [32]. Furthermore, CD169^+^ radiation-resistant tissue-resident macrophages regulate MM cells in the bone marrow via interleukin-6(IL-6) and tumor necrosis factor α (TNF-α) pathway [33]. And adiponectin Adiponectin receptor signaling suppresses TNF-α expression by T cells or myeloid cells [34]. Therefore, we can speculate whether adiponectin changes the biological effect of MM cells by affecting the inflammatory factors. The correlation between the effects of adiponectin receptors and MM progression remains to be studied. Interestingly, adiponectin was found to be downregulated by MM cells themselves via the blockade of TNF-α [8]. It is suggested that adiponectin interacts with myeloma cells to alter the microenvironment of the disease. In addition, it is well known that MM cells enhance the process of osteoclastogenesis and bone resorption while suppressing osteoblast cells differentiation, leading to systemic bone destruction with rapid bone loss [35]. Some studies have paid attention to the association between adiponectin and bone disease. Serum level of adiponectin correlated with markers of bone disease, such as OCN, CTX, and PINP. *In vitro* study, the anti-osteolysis effects of adiponectin may be explained by the inhibition of osteoclasts via the mTOR signaling pathway [36]. In murine models of MM, adiponectin suppressed nerve growth factor, which is thought to be associated with bone pain [37]. Adiponectin may have a positive effect on myeloma bone disease. In summary, adiponectin exerts its antitumor effects in MM by promoting cell apoptosis, causing cell cycle arrest, inhibiting osteolysis, and possibly modulating cytokines in the bone marrow microenvironment (Table 1).

**TABLE 1:** Adipokines and their molecular pathways and functions in MM.

Adipokine	Receptors	Signaling pathways	Functions in MM	References
Adiponectin	AdipoR1 and AdipoR2	PKA (+), AMPK(+)	Promote apoptosis of MM cells	[27]
			Induce cell cycle arrest of MM cells	
			Inhibit the differentiation of osteoclasts	[30, 31]
Leptin	Leptin receptor	STAT-3 (+) Jak/Stat3	Promote proliferation of MM cells	[9, 32]
			Inhibit apoptosis of MM cells	[32, 33]
			Regulate anti-tumor immunity	[34–36]
Visfatin	Insulin receptor	Jak/Stat3	Promote MM cells proliferation	[37]
			Inhibit apoptosis of MM cells	[38, 39]
			Downregulate drug sensitivity of MM cells	[40]
Resistin	CAP1, TLR4, ΔDCN, ROR1	NF-κB, PI3K/Akt	Inhibit apoptosis of MM cells	[41]
			Promote drug resistance	
Apelin	APJ	Unknown	Elevated in patients with MM	[42]
Chemerin	CMKLR1, CCRL2, GPR1	Unknown	Elevated in patients with MM	[43]

CAP1, Adenylyl cyclase-associated protein 1; TLR4, Toll-like receptor 4; ΔDCN, An isoform of decorin; ROR1, Receptor tyrosine kinase-like orphan receptor 1; APJ, apelin-angiotensin receptor-like 1; GPR1, G Protein Receptor 1; CMKLR1, chemerin chemokine-like receptor 1.

These beneficial effects of adiponectin have promoted research on adjuvants that mimic adiponectin or the adiponectin receptor agonist to treat MM. L-4F is an apolipoprotein mimetic peptide that can upregulate the adiponectin level [38]. Moreover, L-4F affected inhibiting the progression of MGUS to MM [31]. In addition, L-4F, downregulating the expression of nerve growth factor (NGF) and IL-6, has a positive effect on destructive osteolytic bone disease [37]. The adiponectin receptor agonist adipoRon can inhibit MM by reversing the effect of TNF-α and IL-6 [37]. Since it was found that adiponectin-induced apoptosis in MM cells may be achieved by downregulating acetyl-CoA-carboxylase. 5-(tetradecyloxy)-2-furan carboxylic acid (TOFA), an acetyl-CoA-carboxylase inhibitor, was found to inhibit fatty acid synthesis and also can inhibit MM cell proliferation [32]. Taken together, the anti-myeloma effect of adiponectin may be a promising target candidate to start with the new area of MM.

#### Leptin

Leptin was the first adipokine to be identified in 1994 and was shown to be expressed in adipose tissue as a secreted 16 kDa polypeptide [39]. Leptin binds to the leptin receptor to exert pleiotropic effects, such as energy homeostasis, metabolism, hematopoiesis, and immunomodulation [40, 41]. A clinical study conducted by Considine et al. showed that serum leptin concentrations in obese subjects were higher than the normal weight subjects [42]. Emerging findings have demonstrated leptin levels in serum samples related to various cancers. Serum leptin level was higher in colorectal cancer patients than in healthy controls [43]. Similar results were found in breast cancer, prostate cancer, non-small cell lung cancer, and bladder cancer [44–47].

The assays of leptin levels in MM patients presented contradictory results. A study including 14 MM patients and 25 healthy controls demonstrated that the serum level of leptin was upregulated in the MM group (22.6 ± 14.7 ng/mL) compared to the healthy control (10.3 ± 7.6 ng/mL) [48]. Similarly, another research between newly diagnosed MM and healthy controls revealed higher serum leptin levels in MM patients [49]. A meta-analysis was conducted by Liu et al. in 2021 to analyze seven studies with 406 MM patients and 530 controls. They demonstrated higher leptin concentrations in MM patients than in controls (SMD = 0.87, CI = 0.33–1.41, z = 3.14, *p* = 0.002) [50]. Alexandrakis et al. examine examined leptin levels in the serum of 62 MM patients, according to the established Durie and Salmon criteria, they divided patients into stage I (*n* = 13), stage II (*n* = 22), stage III (*n* = 27). There was no significance according to each group. The increased leptin levels were not associated with the progression of MM [51]. These results indicate that leptin levels are increased in MM patients. However, Hofmann et al. investigated the serum levels of leptin in 174 patients (10.01 ± 2.64 ng/mL) and 348 controls (9.6 ± 2.71 ng/mL) between 1993 and 2001 in the US and found no significant difference (*p* = 0.78) [52].

The potential mechanisms of leptin on the development of MM could be largely as follows:The potential mechanisms of leptin in the development of MM could be as follows: (1) The pronounced proliferative response induced by leptin. Researchers cocultured MM cell line (RPMI-8226) with adipocytes, MM cells proliferated faster and displayed increased leptin protein level via pSTAT3/STAT-3 signaling [9]. Whether leptin promotes proliferation-mediated STAT3 signaling in RPMI-8226 must be verified by knockdown or by inhibiting leptin expression. Using the cell lines U266 and H929, researchers observed that leptin could promote proliferation, and they also found phosphorylated AKT and STAT3 proteins were increased when upregulated leptin [53]. (2) Leptin may promote MM development by inhibiting apoptosis. Leptin was shown to induce an upregulation of BCL-2 expression and the inhibition of caspase-3 activation [53]. It also can promote the expression of autophagic proteins via Jak/Stat3 pathway and then play an anti-apoptosis anti-apoptotic role in MM cells [54]. (3) Leptin as a regulator of anti-tumor immunity, may contribute to MM oncogenesis. Based on previous studies, we have known that invariant natural killer T (iNKT) cells with the effect of anti-tumor immunity were decreased in MM [55, 56]. Favreau et al. used the murine 5T33MM model, a pre-clinical immunocompetent model mimicking human MM disease, to investigate the leptin levels and expression of the leptin receptor. They found that the leptin level and expression of leptin receptors on iNKT cells were increased obviously. Similar results were obtained in the MM patients. *In vitro*, they coculture the MM cells with iNKT cells and found the IFN-γ production was inhibited. This effect can be reversed by leptin receptor antagonism [57]. Using a leptin-deficient mice model, Wang et al. found leptin increased CD8+T cell exhaustion and upregulated PD-1 expression, which impairs anti-tumor immunity [58] (Table 1).

The results of studies conducted to explore the associations between MM and leptin have been inconsistent and limited. Taken together, the vast majority of the above studies have concluded that leptin is highly expressed in MM. Myeloma types, different stages, risk stratification, and tumor heterogeneity may be responsible for the inconsistent results. Additionally, sex hormone secretion and sleep time could also affect leptin changes [59, 60]. Lastly, leptin levels are more influenced by the amount of subcutaneous fat than BMI [61]. Future clinical studies and additional mechanism studies of leptin in MM are needed further to clarify leptin’s alteration of the bone marrow microenvironment.

#### Visfatin

Visfatin, also known as nicotinamide phosphoribosyltransferase (NAMPT), was initially discovered as a protein for the differentiation of B cells. Named for pre-B-cell colony-enhancing factor (PBEF) [62]. In the intracellular, its function is mainly as the rate-limiting enzyme in NAD^+^ biosynthesis [63]. In the extracellular environment, it was thought to be an adipokine secreted by adipose tissue [64]. Visfatin is highly enriched in visceral fat and found in bone marrow, liver, kidney, and other tissues [62]. The main physiological functions are regulating metabolism, pro-inflammatory, and immune modulation [64]. Serum concentrations of visfatin were significantly higher in overweight and obedity obesity subjects when compared to normal weight controls [65]. Multiple studies conducted in the past decades investigated that visfatin was involved in the progression of different cancer types [66–69].

In MM, visfatin was shown to be elevated in 39 patients when compared with age-matched 20 healthy controls (102.76 ± 90.41 ng/mL vs. 22.55 ± 21.41 ng/mL; *p* < 0.001) [36]. Among some *in vitro* experiments, studies have tried to explore the mechanism of visfatin in MM researchers have attempted to investigate the mechanism of visfatin in MM. In one study, researchers using small interfering RNA (si-RNA) silenced the expression of visfatin then the growth and proliferation of MM cells were inhibited [70]. It was suggested that visfatin might upregulate NAD^+^ to supply energy for MM cells’ growth and survival. Apart from that, researchers also found that visfatin suppress apoptosis of MM cell depending on autophagy [71, 72]. Furthermore, visfatin can downregulate the sensibility of MM cells to bortezomib [73] and reduce the efficacy of anti-CD38 immunotherapies in MM [74]. Finally, in the SCID-rab model, inhibiting the expression of visfatin can suppress osteoclast formation and activity [75]. These studies suggest that visfatin regulates MM cells’ proliferation, apoptosis, drug sensitivity, and bone metastasis.

The research on visfatin as a promising target for treatment has yielded some results. FK866, an inhibitor of visfatin, enhanced the effect of bortezomib and reduced drug resistance. The mechanism of FK866 included activation of pro-apoptosis proteins, reduction of intracellular NAD^+^, and inhibition of angiogenesis [73].

#### Resistin

Resistin was discovered in 2001 and named for its function in promoting insulin resistance and glucose intolerance [76]. Resistin is secreted from white adipose adipose tissue in mice, whereas synthesized from monocytes and macrophages in humans. It is highly expressed in bone marrow [77]. Resistin is involved in insulin resistance, inflammation, immunoregulation, and cancer development [78]. Several studies have demonstrated that resistin can promote proliferation, associate with angiogenesis, regulate the epithelial to mesenchymal transition, and stimulate metastasis in various solid tumors [79–83]. Studies have yielded a positive correlations between serum levels of resistin and BMI [84]. Resistin has been shown to improve the survival and stemness of human breast cancer cells by activating STAT3 signaling [85]. At the neuronal levels, resistin bind to TLR4 receptors inducing the activation of AKT, NF-κB, and MAPK pathway and promoting insulin resistance [86].

Regarding its role in multiple myeloma, one case-control study showed that serum resistin was lower in MM patients (*n* = 73, 9.4 ± 5.0 ng/mL) compared to gender and age-matched healthy controls (*n* = 73, 15.9 ± 6.8 ng/mL, *p* < 0.001) [87]. A nested case-control study involving 178 MM patients and 358 controls showed lower resistin levels in male MM patients (5.2 (3.93–6.46) ng/mL) than controls (5.82 (4.44–7.33) ng/mL, *p* = 0.006) [88]. In contrast, using the primary myeloma cells and myeloma cell lines, Pang et al. have found that resistin suppressed caspase cleavage to promote drug resistance through the NF-κB and PI3K/Akt pathways. Besides, they demonstrated that resistin increased the expression of ATP-binding cassette (ABC) transporters and induced the ATP-driven efflux of chemotherapy drugs. *In vivo* mice model, they validated that resistin plays an anti-apoptosis anti-apoptotic role by annexin Ⅴ binding assay and *in situ* TUNEL assay [89]. Interestingly Reseland et al. showed that the level of resistin had no significant difference between the newly diagnosed MM patients and controls [49]. Pooled analysis of 367 MM patients and 524 controls showed no significant difference in circulating resistin levels (SMD = −0.08, 95%CI = −0.55 to 0.39, *p* = 0.73) [50].

Above all, the role of resistin in MM is polarized. Some studies demonstrated it is a protective adipokine; Pang et al. considered resistin is bad for MM through promoting drug resistance. More studies have shown no discrepancy in the level of resistin between MM patients and controls. The discrepancy in the role of resistin in MM could be explained as follows: First, the secretion of resistin is associated with the dietary approaches, BMI, and the level of glucose [90]. *In vitro* experiments were not affected by the above factors. Second, the different types, stages, and risk stratification may be responsible. However, the exact relationship between resistin and MM warrants further investigation.

#### Apelin

There are two ligands of apelin-angiotensin receptor-like1 (APJ). One is elabela/toddler (ELA), the other is apelin [91, 92]. Apelin also is an adipokine with several functions in many physiological and pathological processes, such as angiogenesis, fluid homeostasis, food intake, and metabolic regulation [93, 94]. The obese subjects presented a higher serum concentration of apelin than controls. The obese subjects (BMI ranged from 31 to 34 kg/m^2^) presented a higher serum concentration of apelin than age-matched controls (BMI ranged from 23 to 24 kg/m^2^) [95]. It was demonstrated that apelin had important effects on malignant diseases. The apelin and apelin receptor systems regulate autophagy and apoptosis [96–98]. Apelin was shown to be elevated (1.99 ± 1.1 ng/mL vs. 0.42 ± 0.16 ng/mL) in patients with MM (*n* = 29) when compared with healthy controls (*n* = 19). Furthermore, the level of apelin may associate with angiogenesis [99]. Some gains have been made in the mechanism of the affection of apelin in several cancers. The exact relationship between apelin and MM warrants further investigation. However, clinical studies with larger sample sizes are needed to support the correlation between MM and apelin.

#### Chemerin

Chemerin was first described as a chemoattractant agent, promoting the chemotaxis of leukocyte populations [100]. The highest expression of chemerin has been detected in white adipose tissue, liver, and lung [101]. Then chemerin is considered an adipokine involved in inflammation, adipogenesis, immunity, and energy metabolism [102]. A growing body of human experimental data indicated that serum chenerin chemerin levels are elevated in obese patients [103]. Increased chemerin levels have been found in gastric, colorectal, and pancreatic cancer [104–106]. The pro-tumor mechanism of chemerin is to promote the proliferation and migration of tumor cells [107, 108]. Westhrin et al. determined the serum levels of chemerin between MM patients (*n* = 122) and healthy controls (*n* = 58). They found chemerin serum levels of MM patients was higher than healthy controls (199.2 ± 88.2 ng/mL vs. 156.5 ± 52.5 ng/mL, *p* < 0.001). It was also shown that the levels of chemerin were associated with the stage of MM [109]. A study has shown that chemerin suppressed the differentiation of osteoblast [110]. It is not known whether chemerin can also play similar roles in myeloma bone disease. As a relatively new adipokine, data on chemerin involvement in MM are sparse. Nonetheless, the clinical evidence demonstrated that chemerin is increased in MM patients, which indicated that possibilities for chemerin as a biomarker should be further explored [109].

## Conclusion

As MM is the second most common form of hematological malignancy and the incidence of obesity is increasing, the need for awareness of the association between obesity and MM development and progression is evident. The correlation between obesity and MM has gained significant attention in recent times. Emma V. Morris and Claire M. Edwards have discussed the association between adiposity, adipokines, and MM [111]. Our review includes new publications from the last 5 years that explore the relationship between MM and new adipokines, including apelin and chemerin. Furthermore, we have also included recent clinical studies that examine the levels of specific adipokines in MM patients, such as leptin. Additionally, we have delved into more in-depth investigations of the roles that visfatin and resistin play in MM. Lastly, the effect of adipokines on the immune microenvironment of MM is a new research highlight.

In this review, we have briefly summarized recent work on representative adipokines linked to MM initiation and progression (Table 2). First, the circulating levels of adipokines showed discrepancies when compared with control groups. Adipokines may be helpful as a biomarker for MM. In addition, adipokines such as adiponectin, leptin, and visfatin can regulate several aspects of myelosis, including promoting MM cell survival and creating a pro-tumorigenic environment for MM. Lastly, there is emerging evidence from *in vitro* studies that showed adipokines interventions could positively affect disease course.

**TABLE 2:** Clinical studies of adipokines in MM.

Author, year	Sample source	Study design	Case			Control			*p*
			Mean	SD	N	Mean	SD	N	
Adiponectin(ug/mL)									
[30]	Serum	Pooled investigation	11.5	No value	624	12.8	No value	1,246	0.001
Leptin(ng/mL)									
[48]	Serum	Case-control Study	22.6	14.7	14	10.3	7.6	25	<0.01
[52]	Serum	Case-control Study	10.01	2.64	174	9.6	2.71	348	0.78
Visfatin(ng/mL)									
[36]	Serum	Case-control Study	102.76	90.41	39	22.55	21.41	20	<0.01
Resistin(ng/mL)									
[87]	Serum	Case-control Study	9.4	5.0	73	15.9	6.8	73	<0.001
Apelin(ng/mL)									
[99]	Serum	Case-control Study	1.99	1.1	29	0.42	0.16	19	<0.001
Chemerin(ng/mL)									
[109]	Serum	Case-control Study	199.2	88.2	122	156.5	52.5	58	<0.001

However, steps forward are hampered by the variation in study outcomes, such as resistin hindered by the inconsistencies in study outcomes, such as resistin. The large heterogeneity partly explains such a phenomenon in MM patients. Based on these achievements, future work should better address as follows: (1) Conducting more clinical and cohorts researches on circulating adipokine levels and the correlation with known biomarkers of immunotypes, bone destruction, and progression. (2) The mechanisms of adipokines affect myelosis and elucidate the pathways of adipokines effects and underlying mechanism in the development of MM. (3) Find potential therapeutic implications target adipokines to halt MM. Taken together, adipokines are promising candidates both for novel pharmacological treatment strategies and as diagnostic tools, provided that we can develop a better understanding of the function and molecular targets of the more recently discovered adipokines.
